# Wood-Veneer-Reinforced Mycelium Composites for Sustainable Building Components

**DOI:** 10.3390/biomimetics7020039

**Published:** 2022-03-31

**Authors:** Eda Özdemir, Nazanin Saeidi, Alireza Javadian, Andrea Rossi, Nadja Nolte, Shibo Ren, Albert Dwan, Ivan Acosta, Dirk E. Hebel, Jan Wurm, Philipp Eversmann

**Affiliations:** 1 Department of Experimental and Digital Design and Construction, University of Kassel, 34127 Kassel, Germany; rossi@asl.uni-kassel.de (A.R.); nadjanolte@uni-kassel.de (N.N.); eversmann@asl.uni-kassel.de (P.E.); 2 Chair of Sustainable Construction, Karlsruhe Institute of Technology, 76131 Karlsruhe, Germany; nazanin.saeidi@kit.edu (N.S.); dirk.hebel@kit.edu (D.E.H.); 3 Arup Deutschland GmbH, 10623 Berlin, Germany; albert.dwan@arup.com (A.D.); ivan.acosta@arup.com (I.A.); jan.wurm@kuleuven.be (J.W.); 4 Design and Engineering of Construction and Architecture, KU Leuven, 1030 Brussels, Belgium

**Keywords:** mycelium, bio-composites, bio-fabrication, digital fabrication, additive manufacturing, ultrasonic welding, wood printing, circular construction, robotic fabrication, reinforced composites

## Abstract

The demand for building materials has been constantly increasing, which leads to excessive energy consumption for their provision. The looming environmental consequences have triggered the search for sustainable alternatives. Mycelium, as a rapidly renewable, low-carbon natural material that can withstand compressive forces and has inherent acoustic and fire-resistance properties, could be a potential solution to this problem. However, due to its low tensile, flexural and shear strength, mycelium is not currently widely used commercially in the construction industry. Therefore, this research focuses on improving the structural performance of mycelium composites for interior use through custom robotic additive manufacturing processes that integrate continuous wood fibers into the mycelial matrix as reinforcement. This creates a novel, 100% bio-based, wood-veneer-reinforced mycelium composite. As base materials, *Ganoderma lucidum* and hemp hurds for mycelium growth and maple veneer for reinforcement were pre-selected for this study. Compression, pull-out, and three-point bending tests comparing the unreinforced samples to the veneer-reinforced samples were performed, revealing improvements on the bending resistance of the reinforced samples. Additionally, the tensile strength of the reinforcement joints was examined and proved to be stronger than the material itself. The paper presents preliminary experiment results showing the effect of veneer reinforcements on increasing bending resistance, discusses the potential benefits of combining wood veneer and mycelium’s distinct material properties, and highlights methods for the design and production of architectural components.

## 1. Introduction

In the past decades, the construction industry has been challenged by the rapidly increasing population and the proportional demand in housing and construction material supply [1]. Concurrently, the excessive energy used, the pollution and the waste generated to produce traditional building materials, such as steel, cement and plastics, impose severe environmental challenges [2]. The majority of greenhouse gas (GHG) emissions results from the processing of materials that are commonly used in the construction industry [3]. The diminution of natural resources and the growing recognition of climate change have been encouraging researchers and companies to seek sustainable alternatives to the currently used materials [4]. The 4R concept of Reduce, Reuse, Recycle and Recover has been increasingly becoming more prevalent to reduce waste and promote circular economy models within industries.

Growing biological materials using plant-based waste from industries can be a potential solution [5]. Among these, the development of bio-based composite materials from mycelium has been introduced recently and could potentially transform the construction sector. Indeed, mycelium-based bio-composites could support the transition towards the utilization of the available organic waste resources by binding them through the mycelium network, further facilitating the development of sustainable and circular alternatives to energy- and resource-intensive construction materials and building products. 

Mycelium has been proven to deliver a range of properties significant to construction, from good acoustic to mechanical properties, including compressive strength, while being a renewable and low-carbon alternative material with relatively good fire-resistance properties [6]. However, one of the major limitations for its application within the construction industry is caused by its low resistance to tension and bending [7]. On the other hand, wood has been known for centuries for its high structural performance. It is an inherently tension-resistant material due to its fiber arrangement [8]. Therefore, this research aims at combining the advantages of each material: exploiting the intrinsic properties of mycelium and wood veneer, and exploring the development of novel, 100% bio-based mycelium-wood veneer composites with improved mechanical properties. 

We explore two methods for increasing material strength: compression with heat and pressure, and the integration of topologically designed reinforcement within the mycelium matrix. While compression improves material strength and Young’s modulus by increasing the density of the material [9], an embedded veneer lattice in mycelium is expected to increase the performance of the composite due to the combination of the compressive strength of mycelium and tensile strength of the internal fiber structure of wood. We investigate these two methods through physical prototyping and testing and compare them in terms of effectiveness, advantages and disadvantages. The possibility of combining the two methods and compressing a veneer-reinforced block is also explored. We develop a hybrid fabrication method suitable for this composite material system and test the samples structurally to assess the effect of compression and reinforcement on composite strength. 

The focus of this research is to explore ways to improve the structural performance of this composite for architectural use cases, while maintaining satisfactory levels of suitable acoustic performance. The intended application is currently planned for interior use; therefore, the water and pest resistance of the resulting composite was not yet studied. The acoustic and fire performance will be subject to further studies.

## 2. State of the Art

### 2.1. Mycelium-Based Composites

The sustainability issues arising from the use of synthetic and non-renewable resins and binders in the engineered wood industry are well known. Thus, new solutions with bio-based resins with a lower environmental impact are being investigated globally [10]. Among the various materials used, mycelium has the potential to be a sustainable and more attractive alternative to most of the available binder matrices. Mycelium is the root part of fungi, composed of filamentous strands of fine white hyphae. When organic substrates, such as wood or natural fibers, are inoculated with specific fungi species, mycelium starts growing by using the substrates’ nutrients [11]. By the time mycelium spreads through the whole substrate, a network structure is developed that binds the discrete particles of the substrate together. Therefore, a range of sustainable and green products can be manufactured in an environmentally friendly way without the need for any adhesives, potentially replacing various energy-intensive building materials. One advantage of mycelium-based composites over traditionally engineered wood-based materials is that they can be recycled or composted at their end of life without any negative impact on the environment [12]. No toxic substances or synthetic components are involved; therefore, mycelium-bound composite materials fit into the model of a bio-circular economy where there is no waste at the end of a product’s lifecycle [6,13]. 

The properties of mycelium-bound materials can be customized to a certain extent by adjusting the parameters of the manufacturing process. A thorough framework of the main parameters influencing mycelium-based composites was presented in various studies recently and identified advantageous material properties, such as low thermal conductivity, high acoustic absorption, and fire protection properties [10,11,12]. However, challenges generally arise from research knowledge gaps [14] that limit the use of these materials only to non-structural or semi-structural applications. 

### 2.2. Mycelium in Architecture

The use of mycelium-based composites as building materials has been explored in recent decades [15]. The most common approach has been to create mycelium building blocks that are assembled into larger structures. However, this application often relies on substructures, and is geometrically quite limiting due to the inherent properties of mycelium that only allows for structures in compression [7,16]. Studies have also been carried out on monolithic mycelium constructions, but these systems require either large scaffolds or extensive reinforcement systems that in most cases take over the structural functions and reduce the mycelium to a surface finishing, rather than a load-bearing material [17].

3D printing mycelium is an emerging research area that uses the mass-customization opportunity as the main research driver. While articulated surfaces created through 3D printing help mycelium growth [18], for large-scale structures, time-efficient additive manufacturing processes have not yet been developed, and the directional dependency of the fabrication method can cause the resulting components to display relatively low structural performance [19].

In order to compensate for the lack of tension and bending resistance of mycelium, research on reinforcing mycelium has been developed recently. Woven textiles, wood fibers, or 3D-printed spatial lattices are among the methods used [20,21,22,23]. However, these studies either heavily rely on manual production, or currently present very limited data about the effects of the reinforcement on the mycelium-based composite strength. For construction applications, to date only mycelium-based foam (MBF) and mycelium-based sandwich composites (MBSC) have been developed and investigated for their properties [24]. The latter uses natural fiber textiles on top and bottom of the components in order to increase bending resistance [25]. 

### 2.3. Additive Manufacturing with Timber

Current additive manufacturing technologies for producing high complexity objects are mainly based on inorganic materials. New processes that allow 3D printing with organic materials have been recently developed [26,27]. For the fused deposition modelling of timber, wood is ground to particles and mixed with various thermoplastics to create continuous printing filaments or pellets [28]. Both these materials cause timber to lose its natural material structure that provides strength, resulting in relatively weak printed structures [29].

**Figure 1:**
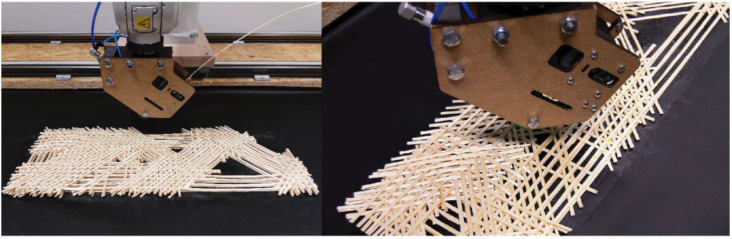
Robotic fiber laying process with processed willow strips from the research project TETHOK—Textile Tectonics for Wood Construction, University of Kassel.

In recent years, researchers proposed a fabrication method for architectural elements, using continuous natural timber fiber filaments through robotic fabrication [30,31]. The aim was to produce structural elements by combining the advantages of continuous fiber-based manufacturing with bio-based materials. This method can achieve highly controlled, sustainable, surface-like [32] and optimized geometries [33], as it can be seen in Figure 1.

### 2.4. Contribution

Combining the previously introduced mycelium composites and wood-based additive manufacturing processes, we propose a novel wood-veneer–mycelium bio-composite and its construction method for carbon neutral, circular building elements. As mycelium has excellent compression properties, but low tension and bending resistance, the integration of tailored continuous wood fibers in the composite is expected to increase the structural capabilities of mycelium-based components, while still being composed of exclusively natural materials. To demonstrate its potential in the context of architecture, we describe the material concept and its production process, and present results regarding its characterization with reinforcement strategies. 

## 3. Materials and Methods

### 3.1. Selection of the Base Materials

#### 3.1.1. Wood Veneer Species

We made a pre-selection of wood species indigenous to Germany, based on their availability at the time of the research, and data from the literature that proved their compatibility with mycelium growth: beech (*Fagus sylvatica*), maple (*Acer pseudoplatanus*), oak (*Quercus robur*), and spruce (*Picea abies*) veneers and willow branches of the genus *Salix americana.* Initial binding tests with these selected species were carried out at the University of Kassel. Maple demonstrated the best wood–wood bond with the selected binding method and was chosen as the reinforcement material. 

H. Heitz Furnierkantenwerk from Melle, Germany supplied FSC (Forest Stewardship Council) certified maple veneer edge-bands that were 12 mm wide and 0.5 mm thick in spools. They are produced by lining up veneer sheets and joining them with fully glued finger joints. Non-woven cellulose-based fleece on one side of the roll is then added with PVAc dispersion glue to ensure that the material does not easily break during application. Due to the commercially available veneer rolls using a small amount of glue for their joining during production, the presented composites are not yet fully bio-based. However, custom veneer rolls made with bio-adhesives could be produced and utilized in future studies. 

#### 3.1.2. Substrates

We made a pre-selection of the substrates based on the availability of the raw materials mainly as waste stream in Europe: hemp fibers, hemp hurds, pine wood sawdust and shavings, and Silvergrass (Miscanthus) shavings. For the purpose of this study, only hemp hurds were used, which were collected from Bafa GmbH (Malsch, Germany), a local wood mill.

#### 3.1.3. Mycelium Species 

The mycelium mother culture of *Ganoderma lucidum* (*G. lucidum*) was purchased from Tyroler Glückspilze (Innsbruck, Austria) in the form of grain spawn and stored at 4 °C for up to four weeks. This selection was mainly made due to the already known faster growth rate on hemp hurds, and its availability in Europe. *Ganoderma lucidum* was grown on hemp hurds and subsequently reinforced with maple veneers to carry out a series of physical and mechanical tests on lightweight and dense veneer-reinforced mycelium-based composites. 

### 3.2. Fabrication

#### 3.2.1. Robotic Wood Fiber Laying

**Figure 2:**
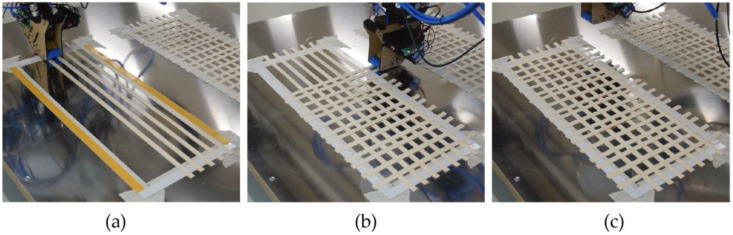
Robotic fiber laying process: (**a**) Fiber laying in direction one; (**b**) Fiber laying in direction two; (**c**) Completed 2D lattice.

We developed a custom fabrication process to lay the continuous wood fibers robotically. The process consisted of the following sub steps: a single wood strip was extruded at a time, and the material was cut when a change in extrusion direction was needed. This allows for complex tool paths, the creation of multi-directional reinforcement patterns and controlled anisotropy. Two approaches can be used for the layering: placing the veneers with the same direction or similar directions at once, then moving on to the next layer (Figure 2); or printing one line from a different direction at a time, which results in a structure with interwoven fibers [31].

**Figure 3:**
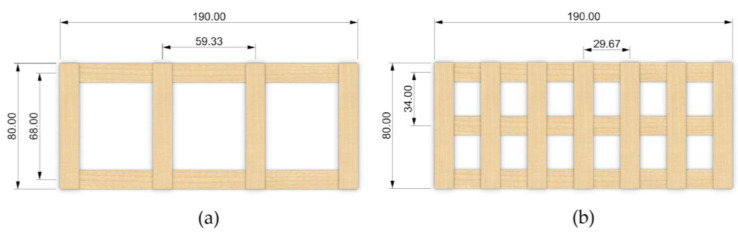
Veneer lattices produced: (**a**) Low-density lattice; (**b**) High-density lattice (dimensions in mm).

We designed two types of 2D veneer lattices to reinforce the mycelium blocks/boards for this study: high- and low-density lattices (Figure 3). While the low-density lattice had two veneer strips in the longitudinal direction and four in the transversal direction, the high-density lattice had three and seven veneer strips, respectively. In each lattice, we placed the veneers to form a frame that was 19 cm × 8 cm in a total of two layers that were perpendicular to each other. The veneer strips were fixed at their ends using double-sided tape during printing. After all strips with the same direction were laid down, the second layer of strips perpendicular to the first layer was added, and the lattices were ready for the ultrasonic welding of the intersection points.

#### 3.2.2. Ultrasonic Wood Welding for Wood–Wood Binding

Precedents of continuous wood fiber laying research have explored synthetic binders, such as UV-curing glue, contact glue and hot melt glue [31]. Since mycelium growth is incompatible with synthetic materials, and the goal of producing a 100% bio-based composite cannot be achieved with the binders investigated to date, it was necessary to research alternative binding methods.

Ultrasonic welding is a common adhesive-free joining method used in many industries, including automotive, electronic, and medical, due to its speed. It is performed by using ultrasonic energy at high frequencies that produce mechanical vibrations, which results in heat due to the friction between the two elements to be joined. Heat melts thermoplastic materials and binds the parts together after cooling [34]. In recent decades, this method has been used to weld thin woo, through heat softening and melting lignin in wood and binding the materials with entangled fibers [35]. Considering that no adhesives are needed for joining, this method was chosen as the wood–wood binding strategy for our custom manufacturing process.

**Figure 4:**
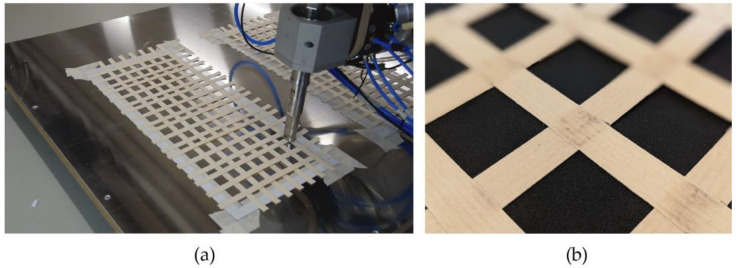
(**a**) Robotic welding process; (**b**) Welded intersection point close-up.

The wood welding was performed with an ultrasonic welding horn, a generator that uses 20 kHz frequency, and a flat-ended sonotrode provided by Weber Ultrasonics (Karlsbad, Germany) mounted on a robotic arm (Figure 4). 

As the veneer rolls have fleece on one side, initial welding tests were made comparing the welds of wood to wood, wood to fleece and fleece to fleece sides. The material was always placed with the wood side facing the welding horn to avoid the fleece from sticking onto the welding horn. Once the robot reached the intersection point to be welded, the pressure was applied by moving the robot arm down in the vertical direction. Then, the welding was performed by a signal of the digital control unit connected to the generator. 

#### 3.2.3. Mycelium-Based Composite Fabrication

##### Substrate Inoculation

Hemp hurds were collected from a local wood mill called Bafa GmbH (Malsch, Germany) and mixed with wheat bran to enhance the growth of mycelium, while calcium sulfate (CaSO₄) was added in a dry condition to adjust the pH of the mixture to the desirable threshold of 5 to 6, suitable for mycelium growth. The mixture was then blended with 60 wt% (weight percentage) of water, and eventually sterilized at 121 °C for 60 min. Subsequently, the mixture was cooled to room temperature before it could be inoculated with the selected *G. lucidum* grain spawn. Once cooled down, it was mixed with 1 wt% of colonized mycelium spawn. The colonized substrate was eventually left in the incubation room at 26–28 °C with 70–80% humidity for two weeks to develop the full mycelium network. 

##### Molding and Sample Preparation 

**Figure 5:**
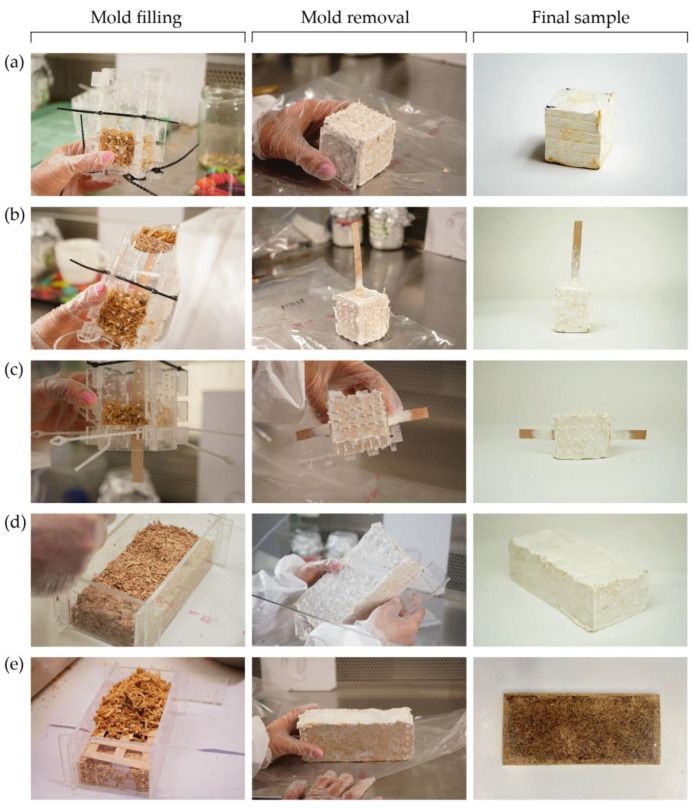
Mycelium composite samples’ production process: (**a**) Compressive strength test cube; (**b**) One-side single veneer pull-out test cube; (**c**) Two-side, middle overlapped veneer with and without welding reinforced cube; (**d**) Lightweight block with and without low- and high-density lattices; (**e**) Pressed board with and without low- and high-density lattices.

After two weeks of substrate colonization in the incubation room, the samples were taken out and transferred into molds prepared for compression, pull-out, and flexural tests. Following the filling of the molds with colonized substrates, they were transferred to the incubation room with similar conditions to the previous phase. The molds were kept there for an additional 3–6 days until the mycelium network was observed to have covered the substrate surface. Then, the mold was removed, and the samples were left in the incubation room for another 3–5 days to expedite the growth of the mycelium network inside and on the surface of the samples with better aeration. The samples´ preparation process and the final mycelium-based composites for each type of test are shown in Figure 5. 

For compression and pull-out tests, we prepared cubes of 5 × 5 × 5 cm^3^. A moistened and sterilized maple veneer strip with a length, width and thickness of about 16 cm, 1.2 cm, and 0.05 cm, respectively, was then placed in the center of the mold, parallel to one mold side for the pull-out tests. We prepared three types of pull-out samples: a series with a single veneer strip penetration of up to 75% of the height of the cubes, samples with unwelded overlapped veneer extending from both sides of the cubes, and lastly samples with overlapped and welded veneer. For the last two series of the above-mentioned samples, we placed the overlapped section of the veneer strips in the center of the cubes. The tests aimed to determine the interfacial shear strength between the mycelium matrix and veneer, and to evaluate the bonding mechanism that was developed at the interface of the veneer and mycelium matrix. 

Flexural samples were prepared in molds of 19 cm × 8 cm × 7 cm, with and without veneer lattices (Figure 5d,e). First, we filled up half of the height of the mold with the colonized substrates before placing a veneer lattice, and afterwards finished filling up the rest of the mold with substrate. It was ensured that the density of all the samples would stay the same throughout the sample preparation. We used two types of veneer lattices for this study: high- and low-density lattices. Given the lack of prior research on the use of veneer reinforcement for mycelium-based composite materials, the size of the samples was chosen to suit the available testing facilities, while necessary references to ASTM and European standards were made. For comparison purposes, we prepared a series of flexural test samples with two layers of low-density veneer lattices embedded: one lattice at the top and one at the bottom of the molds with a 10 mm distance from the surface of the substrate. Further details are provided in Section 3.2.4. 

##### Post-Processing 

Once the growth cycle was completed, the samples were transferred to a drying oven and kept there at a temperature range of 60–70 °C for 2–3 days. The samples were weighed regularly during this period to ensure their weight was stabilized. When no change was observed, they were removed from the oven, and their final density was measured. 

Compression and pull-out test samples were directly tested after drying, while the flexural test samples with and without veneer lattices were prepared for an additional pressing process to produce dense mycelium-based composites (DMC) as per the procedure explained in an earlier study [11]. The flexural test samples were placed in a hot press compression molding machine and pressed at a temperature of 120 °C, with the pressure set to 10 MPa for a duration of 15 minutes. The compressed samples were then moved to an oven with a temperature of 40 °C for 12 to 24 h to adjust to the room temperature and avoid any thermal stress shock within the samples. 

#### 3.2.4. Testing

##### Tensile Tests

Ten maple veneer strips were tested in order to determine the tensile strength of the reinforcement material used for this study. Each end of the veneer strip was fixed to the grip of a UTM with a 30 kN HBM load cell attached, and pulled by applying 1 N with a loading rate of 10 mm/min. 

Similarly, the weld strength was also investigated through tensile tests. Two maple veneer strips that were 10 cm long were overlapped along their grains (in the same axis) and on the end points with an area of 1.2 cm × 3 cm, in which the 1.8 cm from the center of the overlap was welded. Twenty samples were prepared, and the ends were fixed to the grip of the testing machine with the same setup and pulled apart by applying 1 N with a loading rate of 10 mm/min.

A testing standard specific to wood veneers was not found. However, for the climate conditions of the testing, there are numerous standards, such as DIN 52377 (Testing of plywood—Determination of modulus of elasticity in tension and of tensile strength), DIN EN 302-x and DIN EN 205 (Adhesives—Wood adhesives for non-structural applications—Determination of tensile shear strength of lap joints) for wood, and DIN EN ISO 291 (Plastics—Standard atmospheres for conditioning and testing) for plastics with very similar conditions that can be considered as a baseline. Therefore, the climate recommendations from these standards were taken as the reference and the tests were carried out in circa 23 °C and at 50% humidity. While the welded samples were stored in 20 °C and at 60–65% humidity prior testing, single veneer strips were tested immediately in the recommended testing room conditions. The test setup was designed as per recommendations given by the DIN EN 205 testing standard, while the sample shape had to be adapted due to the material restrictions. Other specifications, such as clamping length and temperature, were followed.

Since almost all the welded samples demonstrated material failure rather than joint failure (see Section 4.2.1), tensile strength was evaluated instead of shear strength. The following formula was used for the calculation:

(1) $$\sigma_{t}=\frac{F_{max}}{bt}$$

where *F_max_* stands for the maximum load in N measured by the UTM at the failure, and *b* and *t* represent the specimen width and thickness in mm, respectively.

##### Compression Tests

The compression test samples had an average density of 145 kg/m^3^, which places them in the range of flexible polyurethane foam products, due to their soft texture and low density. Given the lack of standard testing methods for lightweight mycelium-based materials, ASTM D3574:2017 (Standard Test Methods for Flexible Cellular Materials—Slab, Bonded, and Molded Urethane Foams), which is a commonly used standard for testing flexible cellular foam materials, was used as the reference for the testing and evaluation of the compressive properties. The samples were tested using a Universal Testing Machine (UTM) with a 5 kN HBM load cell (HBK, Germany). The cubes were compressed at a rate of 0.5 mm/min and tested until failure. The compressive strength and elastic modulus were calculated using the following formula:

(2) $$\sigma_{c}=\frac{F_{max}}{bd}$$

where *F_max_* is the failure load in N recorded during the test, and *b* and *d* are the width and depth of the cubes in mm, respectively. The elastic modulus was calculated using the slope of the stress–strain curves obtained from each individual test. 

##### Pull-Out Tests

The bond between the veneer and mycelium matrix can be assessed with the pull-out tests, similar to the methods employed to measure the reinforcement-concrete matrix bond in steel-reinforced concrete elements. There are multiple testing standards with similar scenarios where the reinforcement (veneer strips in this study) is embedded in concrete. Using a UTM, the reinforcement is pulled out by applying a tension force with a defined loading rate, while the sample is restrained to avoid its movement. However, a testing standard for the exact type of material combination of timber and mycelium does not yet exist, since it is a novel composite material. Therefore, testing standards for other materials had to be followed. We selected the procedures explained in RILEM technical recommendation (RC6 Bond test for reinforcement steel. 2. Pull-out test) and ASTM D7913:2020 (Standard Test Method for Bond Strength of Fiber-Reinforced Polymer Matrix Composite Bars to Concrete by Pullout Testing) as the most relevant standards for this study. Thus, we designed the test setup as per the recommendations given by these two testing standards and made the necessary modifications to suit the available testing machines.

The interfacial shear strength (IFSS), or the bond strength, was measured by using a 5kN HBM load cell attached to a Universal Testing Machine (UTM). As explained earlier, three types of pull-out tests were performed in this study. For the pull-out tests where the veneer strip was extended only from one side of the cubes, the veneer strip was fixed to the grip of the tensile test setup and was pulled out on the fixed end. It was ensured that the cube could be held in place to prevent any movement or slipping during the tests. Subsequently, the IFSS was measured using the following formula:

(3) $$\tau =\frac{F_{p}}{2l\left(t+b\right)}$$

where *F_p_* is the pull-out force measured by the machine in N; *t* and *b* are the veneer thickness and width in mm, respectively; and *l* is the embedded veneer length (75% of the cube height) in the mycelium matrix in mm. 

In the case of welded and unwelded overlapped veneers, which extended from the two sides of the pull-out samples, a similar overlapped area of 1.2 cm × 3 cm was used to compare the bonding properties. The veneers were overlapped and then embedded within the mycelium matrix and the two free ends were pulled out with the help of the UTM tensile grips from both sides. 

##### Flexural Tests

To evaluate the flexural capacity of the samples, the recommendations of ASTM D1037:2020 (Standard Test Methods for Evaluating Properties of Wood-Base Fiber and Particle Panel Materials) were adopted. A three-point flexural test was used to find the modulus of rupture and to evaluate the flexural properties, including the elastic modulus in flexure. The support span was set to 140 mm, and a loading rate of 1 mm/min was used for the testing. The lightweight blocks and dense boards with and without veneer lattices were each tested for their flexural properties. The Modulus of Rupture (*MOR*) was calculated using the following formula, while the elastic modulus in flexure was calculated using the stress–strain curves obtained for each sample from the UTM:

(4) $$MOR=\frac{3LF_{max}}{2bt^{2}}$$

where *F_max_* stands for the maximum load in N measured by the UTM at failure, and *b*, *t* and *L* represent the specimen width, thickness, and distance between the support points in mm, respectively. *L* was set to 140 mm for all the samples, given the size of the specimens and the available testing machines. It should be noted that the lightweight blocks and the dense boards had a final size of 18.5 cm × 7.5 cm × 6.5 cm after drying and pressing.

## 4. Results

### 4.1. Fabrication

We successfully developed a custom robotic fabrication process consisting of two steps for this study: robotic wood fiber laying and ultrasonic wood welding. These individual processes were carried out, respectively, using different end effectors mounted on a robot arm. By carefully placing the wood fibers and binding them where necessary, we produced flat lattices made up of two orthogonal layers with this method and used them to reinforce mycelium blocks. Thanks to the mechanical properties and directionality of the material being preserved with this fabrication method, precise control over the reinforcement orientation was achieved.

Despite the geometric freedom of this additive manufacturing method, we encountered some challenges in the production: when placing the veneer, the start and end points of the strips should be fixed. Similarly, when more than one layer is deposited on one point, the strips must be bound together to stay in place until mycelium growth. Since only 2D lattices were produced for this study, this was solved by fixing the end points of the veneers with double sided tape on an aluminum plate. However, to produce more complex geometries with multiple layers, a more robust and local solution needs to be researched. 

As a result of the initial welding tests, the most promising bond was obtained with the sample where the wood side of one veneer was welded to the fleece side of the other. Therefore, all samples were produced, while keeping the wood side on top; two layers of veneer were overlapped on their opposite sides (wood to fleece) and prevented the fleece from sticking to the sonotrode. 

The welds on the intersection points were successful in keeping the lattice together during mycelium growth. However, not every point could be welded at once, or with the same quality due to slight pressure differences caused by using robot motion to apply pressure for welding. The welding setting used for most of the intersection points was 0.5 seconds with 100% amplitude. While this setting performed well in most welds, inconsistencies were observed, with burned welds and welding failures. In our robot setup, we used contact pressure through robot motion, which was not possible to be precisely controlled. This is therefore assumed to be one of the main reasons for welding inconsistencies. With the welding equipment being highly sensitive, if the sensors detect that the target welding time is not reached, or the system uses too much power to weld, the process is interrupted. In further studies, the integration of a force-controlled pneumatic cylinder and motion control system is planned to ensure constant pressure, prevent delays in production, and provide weld consistency.

### 4.2. Testing

#### 4.2.1. Tensile Tests

**Figure 6:**
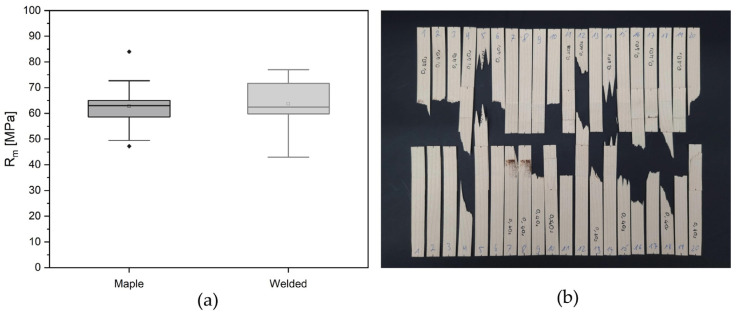
Tensile strength tests: (**a**) Comparison graph of maple veneer’s tensile strength to welded joints’ tensile strength; (**b**) Tested welded veneer samples.

Most samples showed wood tensile failure (Figure 6b), rather than welding area failure, proving that the weld strength is higher than the veneer tensile strength. The average tensile strength of the welded samples was measured as 63.6 ± 10.5 MPa, which is almost the same as the average tensile strength of a single maple veneer strip, 61.95 ± 10.83 MPa, and confirms the previous statement (Figure 6a).

#### 4.2.2. Compression Tests 

The results of compression, pull-out and flexural tests of all samples, together with their densities, are summarized in Table 1.

**Figure 7:**
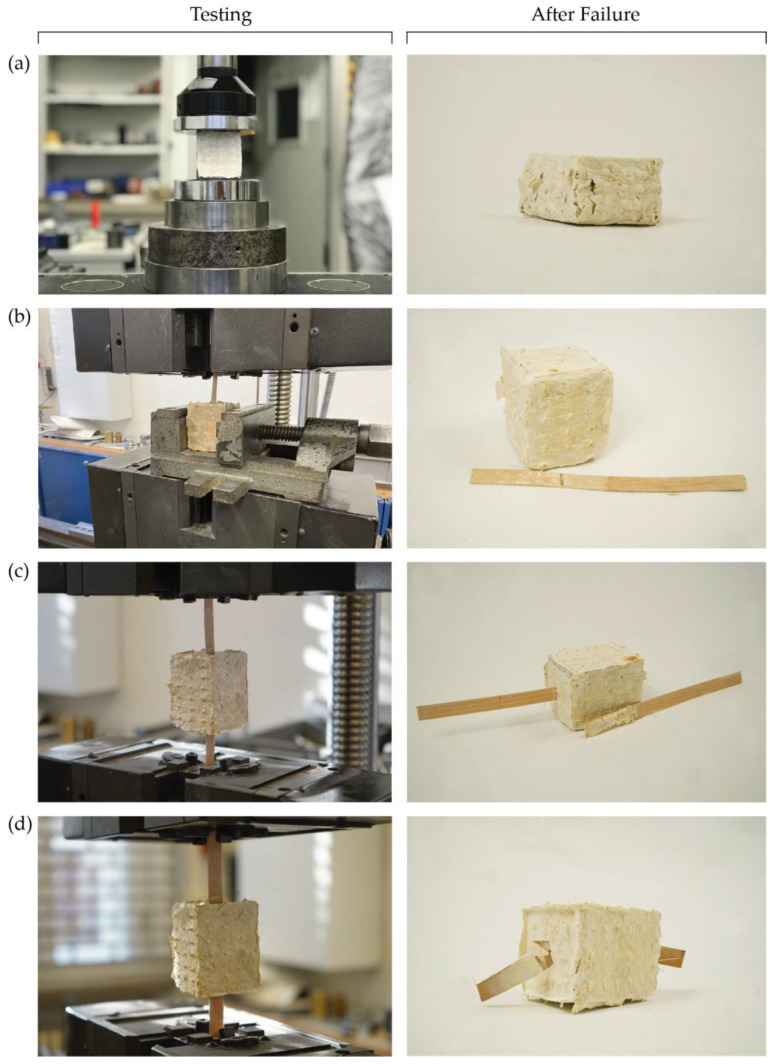
Samples during testing and after failure: (**a**) Compressive strength test; (**b**) One-side veneer pull-out test; (**c**) Two-side unwelded veneer pull-out test; (**d**) Two-side welded veneer tensile test.

The average compressive strength of the samples was 1.2 MPa and the average elastic modulus in compression was measured to be around 4.1 MPa. As it can be seen in Figure 7a, the samples were compressed until reaching 50% of their original height. No cracking or breakage was observed during the tests. The samples showed deformation under compression load and were compressed until the end of the test. It was also observed, when the test continued beyond 50% of the sample’s original height, that higher loads could be achieved. However, given the recommendations of the ASTM D3574, the test continued until the samples were compressed to 50% of their original height and showed similar behavior to conventional foams and flexible cellular materials. 

#### 4.2.3. Pull-Out Tests

The pull-out samples with one strip of maple veneer in the middle showed similar IFSS as those samples prepared with two strips of unwelded maple veneer. Mycelium growth was observed on the maple veneer surface that was embedded within the mycelium matrix (Figure 7b,c). The samples have shown a relatively good bonding: the bond strength between the mycelium matrix and the veneer strips was measured as 0.34 MPa and 0.36 MPa for a single veneer strip and overlapped veneer strips, respectively. The slight increase in the IFSS in samples with two unwelded overlapped veneer strips can be attributed to the better growth of the mycelium network around and between the layers of the veneer strips. A stronger bond was developed in these areas in comparison to the samples with a single strip of veneer where less surface area resulted in a lower mycelium growth density. For both series of samples, clear pull-out of veneers from the mycelium matrix was observed. 

However, for samples prepared with welded overlapped maple veneers, the failure of the veneer strips due to tensile mode of failure was observed, before the veneer could be pulled out (Figure 7d). This can be explained by the higher failure load observed during the tests compared to the other pull-out samples. Furthermore, when the resulting stress is compared with the tensile strength of the maple veneer, it can further validate the hypothesis that the weld strength is relatively higher than the bond strength between the veneer strips and the mycelium matrix (Figure 6). However, further testing is required to find out the effect of mycelium growth combined with welded veneers on improving the bond strength in wood-veneer-reinforced mycelium-based composites. 

#### 4.2.4. Flexural Tests

**Figure 8:**
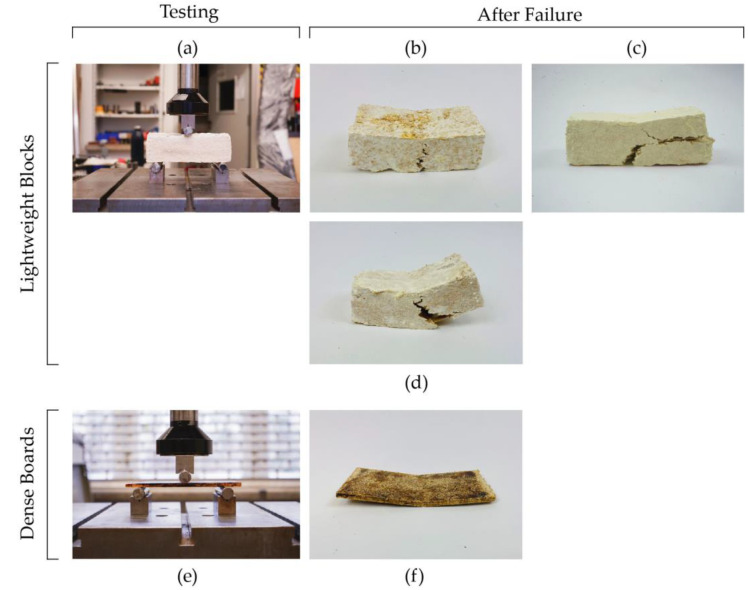
Samples during testing and after failure: (**a**) lightweight block under 3-point flexural test; (**b**) Block without or with low-density lattice in the middle; (**c**) Block with high-density lattice in the middle (shear failure); (**d**) Block with two layers of low-density lattices close to the top and bottom of the block; (**e**) Dense board under 3-point flexural test; (**f**) Dense board after failure without lattice reinforcement.

**Figure 9:**
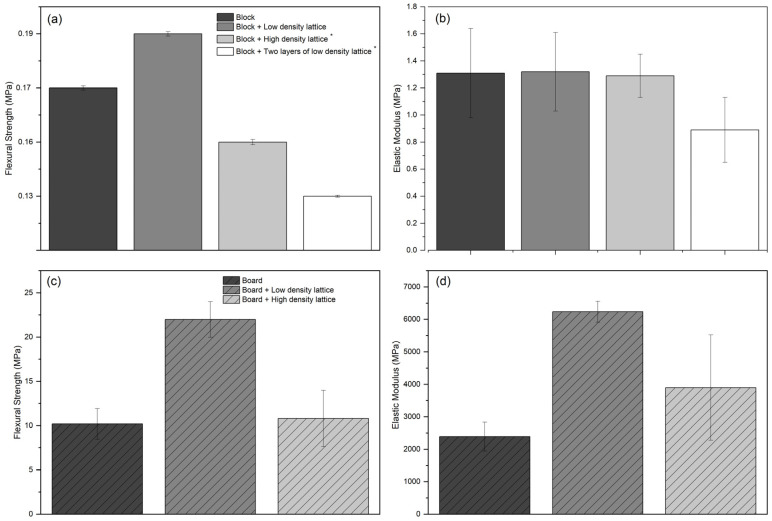
Flexural properties, including strength and elastic modulus: (**a**) Flexural strength of lightweight blocks; (**b**) Elastic modulus in flexure of lightweight blocks; (**c**) Flexural strength of dense boards; (**d**) Elastic modulus in flexure of dense boards. * Shear failure was observed, further explanation is given in the text.

Lightweight samples with one layer of high-density veneer lattice and the ones with top and bottom low-density veneer lattices showed shear mode of failure, while all the other samples, including dense boards with low- and high-density veneer lattices, and samples with no veneer lattices showed flexural mode of failure (Figure 8). The flexural strength of lightweight blocks increased slightly with the addition of one layer of low-density veneer lattice in the middle, compared to non-reinforced blocks. On the other hand, the use of high-density lattices resulted in shear failure and lower flexural strength. Similarly, samples with top and bottom low-density lattices also showed lower flexural strength. The results are summarized Figure 9.

The abovementioned behavior could be explained by the shear failure mode as a result of the potentially lower bonding strength between the veneer lattices and mycelium matrix. High-density lattices decrease the areas where the mycelium network would grow through the lattice holes and connect the two sides of the block divided by the lattice. This would create a weaker interlocking mechanism, which could result in a higher chance of de-bonding when exposed to flexural loads. Furthermore, the lower flexural strength of these samples compared to low-density lattices could also be attributed to the mycelium growth on the veneer lattices. In the case of high-density lattices, more surface area would result in a higher bonding strength between the veneer strips and substrates by forming a stronger mycelium network. However, as was observed from the pull-out tests of samples with unwelded overlapped veneer strips, the bond mechanism was not fully developed and all the samples showed clear pull-out failure, rather than the tensile failure of the veneer strips. Therefore, it is possible to state that weaker bonding areas between the mycelium matrix and the veneer strips increase the chance of de-bonding and interlaminar shear failure. 

Unlike the lightweight samples, dense boards showed clear flexural failure, which indicates a stronger bond mechanism between the veneer strips and mycelium matrix (Figure 8f). Even though no shear failure was observed, similar trends in flexural strength could be observed when the high-density veneer lattices were used in dense boards. No significant increase in flexural strength was detected within dense boards with high-density lattices compared to dense boards with no lattices. However, dense boards with low-density lattices showed a significant increase in both flexural strength and elastic modulus. In general, it was also observed that the increase in density helped to increase the flexural properties when the results of dense boards are compared with lightweight blocks. 

Further testing is required to evaluate the impact of different veneer lattice densities and layouts in combination with different substrate densities on the flexural properties of wood-veneer-reinforced mycelium-based composites. Furthermore, the investigation of veneer placement within the samples along the height of the blocks should also be carried out to explore the bond mechanism developed between mycelium matrix and the veneer lattices with varying densities, and their impacts on flexural properties of the final samples.

Lightweight samples reinforced with either one layer of high-density lattice in the middle or two layers of low-density lattices on top and bottom of the block showed shear failure (Figure 8c,d). However, for lightweight samples reinforced with one layer of low-density veneer lattice and dense boards with low- and high-density veneer lattices, flexural failure was observed as the dominant mode of failure as expected (Figure 8b,f).

## 5. Discussion

The wood fiber laying process used in this study has the potential to become a resource-efficient, rapid production method, with material carefully placed in the structurally required areas. Fiber placement and binding were carried out with two different end effectors, as sequential steps of the process. In order to speed up production, further studies are planned to develop a single tool that can lay wood veneers and at the same time weld the intersections.

The novel wood-veneer-reinforced mycelium composites developed in this study were investigated for their mechanical properties, including compressive strength, pull-out strength and flexural properties. The suitability of 2D veneer lattices as a reinforcement system with welded and unwelded joints was also investigated separately through a series of tensile tests. The results of the investigation of welded joints show that they perform relatively well; in the majority of the tensile tests, no failure in the joints was observed, which indicates that the joints have a higher strength than the veneer itself. The bonding between the veneer strip and mycelium matrix was investigated through a series of pull-out tests and the results show that the bond might not have been developed fully, as most of the single veneer-strip-reinforced cubes showed purely pull-out failure modes rather than any failure in the veneer strip. However, the visual examination after completion of the tests confirmed the growth of mycelium network on the veneer strips. This again validates our hypothesis that the selected mycelium species (*Ganoderma lucidum*) can grow well when combined with the selected veneer species (maple) and hemp hurds as the main substrate. 

Furthermore, the performance of the welded and unwelded joints was investigated through a series of pull-out tests with similar overlapping veneer areas embedded within the mycelium matrix. It was observed that the welded joints outperform the unwelded ones. The results show that, even though the mycelium growth was observed in both cases on the veneers, the interfacial shear strength developed within the unwelded veneer strips and mycelium matrix was lower than the strength of the welded joints. Further investigation on enhancing the mycelium growth on the veneer strip and improving the interfacial shear strength between the mycelium matrix and veneer strip is necessary to achieve a better bonding strength. 

The results of the flexural tests on various samples once again strengthen the hypothesis that the bonding between the veneer reinforcement and mycelium matrix plays an important role in the structural integrity and mechanical properties of these composites. Moreover, compressing the lightweight blocks into dense boards showed a significant improvement in flexural properties as a result of densification, and improved the bending mechanism between the veneer lattice and mycelium matrix. While samples with top and bottom veneer reinforcement did not show any significant increase in the overall flexural properties, samples with one layer of low-density veneer lattice before and after compression showed better flexural performance. The lower flexural strength and elastic modulus measured correspond to the shear mode of failure observed during the tests. Therefore, further investigation is necessary to identify the optimum design of the veneer lattices and to explore the effect of connecting the top and the bottom reinforcement lattices, namely 3D lattices. 

Composed of one bottom and one top 2D lattice connected by an undulating layer of wood veneer, 3D lattice reinforcements could potentially improve the shear capacity of the mycelium composites and provide additional strength and stiffness via the spatial lattice system. Their design would be strictly connected to the design of the base 2D lattice. Following the production of the flat lattice, the layer that gives the structure its depth would be achieved through placing the material diagonally between the two opposite corners of a quadrilateral cell created by the 2D lattice, with a pre-calculated length. More material length would result in more structural depth. 

This system could also achieve surfaces with varying depths through the management of the middle layer’s height and the corresponding non-parallel and non-planar top and bottom surfaces. Similarly, through the gradual cell size modifications along the structure, heterogeneous reinforcement could be achieved. The parameters that can be adjusted on a cell level provide high flexibility, and opportunities for lightweight, optimized reinforcement based on local requirements. 

**Figure 10:**
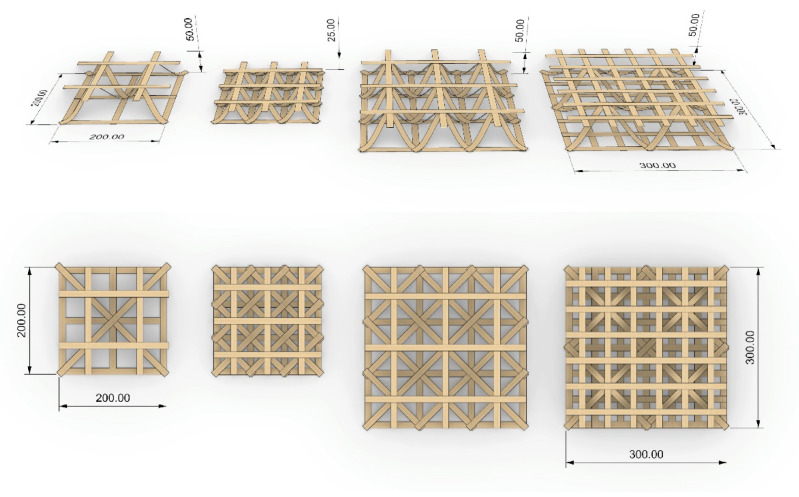
3D lattice layout studies (dimensions in mm).

However, certain limitations must be considered when designing 3D lattices with wood veneers: different veneer thicknesses and species allow for different bending radii. Due to the fibers being oriented in the direction of fiber laying, forcing the material into a very small bending radius would result in the filament breaking. Therefore, the minimum bending radius would be the main determining factor for the component height and as a result, the 3D lattice density. In Figure 10, some 3D lattice design studies based on the bending radius of maple veneer can be seen. 

## 6. Conclusions 

A novel wood-veneer-reinforced mycelium-based composite material was developed for this study as a sustainable and green alternative to traditional building materials with potential applications in the construction industry. Structural testing on physical prototypes was carried out to investigate the fundamental mechanical properties of this novel composite. The test samples were prepared with different variations of veneer lattices as reinforcement systems and tested for compressive strength, bond strength, and flexural properties. The tests provided an initial understanding of the mechanical behavior of the wood-veneer-reinforced mycelium composites in terms of densities, strength and stiffness at material scale. Both strategies, integrating a topologically designed veneer lattice and compression with heat and pressure, proved to be effective methods in increasing the bending resistance of the presented composites. It was shown that the effect of veneer lattices as reinforcement systems is strictly tied to the density and configuration of the lattice. For lightweight blocks, the most promising results were achieved with a single layer of low-density veneer lattice placed in the middle of the mycelium block. This configuration helped to increase the flexural strength of the block slightly (approximately from 0.17 MPa to 0.19 MPa), whereas the high-density lattice and two low-density lattices at the top and bottom of the block resulted in a lower flexural strength (approximately 0.16 MPa and 0.13 MPa, respectively) than that of the unreinforced block itself. The samples with two low-density lattices demonstrated a low flexural strength and elastic modulus and resulted in shear failure. Therefore, 3D lattice systems connecting top and bottom lattices are proposed to avoid shear failure in lightweight blocks for future studies. 

The dense boards with one low-density lattice in the middle demonstrated a similar trend to the lightweight blocks and increased the flexural strength to more than double (from approximately 10 MPa to 25 MPa) of the unreinforced dense boards. On the other hand, the dense boards with one high-density lattice in the middle did not show a significant change in flexural strength compared to the unreinforced dense boards.

When the two methods are compared, dense boards have a better overall flexural strength. The dense boards reinforced with one low-density lattice are the most promising specimens, and would be appropriate for applications that require planar components and higher bending resistance. However, if more complex geometries that do not require high bending resistance are needed, lightweight blocks reinforced with one low-density lattice would be suitable. 

The study provided the fundamental material inputs for the further development of the system at a larger scale. In the next steps, a digital model will be developed to integrate the material properties as design inputs and material constraints; geometrical variations as design variables; and structural Finite Element Analysis (FEA) and acoustic analyses as solvers to evaluate and optimize various design options within one digital computational framework. 

Further studies, including the investigation of the growth compatibility between other wood veneers and mycelium species combined with a range of available organic waste by-products from wood and agricultural industries, will be carried out in the next steps of the research. Additional mechanical testing of the mycelium composites as larger panels reinforced with 3D lattice systems made of veneer are also planned to gain further insights into the materials’ behavior, which will subsequently support the design and development of these composites. The initial results obtained show that wood-veneer-reinforced mycelium composites could be a promising environmentally friendly and sustainable substitute material to conventional building materials with potential applications in the context of architecture.

## Figures and Tables

**Table 1:** Summary table for physical and mechanical properties of mycelium-based composites with and without veneer lattices.

Test	Sample	Density (kg/m^3^)	Strength (MPa)	Elastic Modulus (MPa)
Compressive strength	Cube	145 ± 14	1.2 ± 0.12	4.10 ± 0.67
Pull-out strength	Cube + one-side veneer		0.34 ± 0.04	NA
	Cube + two-side of unwelded veneer		0.36 ± 0.1	NA
Tensile strength	Cube + two-side of welded veneer		30.6 ± 3.6	NA
Flexural strength lightweight	Block	140 ± 8	0.17 ± 0.04	1.31 ± 0.33
	Block + low-density lattice		0.19 ± 0.04	1.32 ± 0.29
	Block + high-density lattice *		0.16 ± 0.05	1.29 ± 0.16
	Block + 2 layers of low-density lattice *		0.13 ± 0.02	0.89 ± 0.24
Flexural strength dense	Board	1180 ± 75	10.2 ± 1.73	2390.95 ± 444.91
	Board + low-density lattice		21.99 ± 2.01	6236.22 ± 322.2
	Board + high-density lattice		10.81 ± 3.18	3900.2 ± 1621.9

* Flexural failure was not observed for these samples; the failure mode was shear as explained in the text.

## Data Availability

Data are available upon request.
